# Impact of Insomnia Symptoms on the Clinical Presentation of Depressive Symptoms: A Cross-Sectional Population Study

**DOI:** 10.3389/fneur.2021.716097

**Published:** 2021-08-09

**Authors:** Yun Ho Choi, Kwang Ik Yang, Chang-Ho Yun, Won-Joo Kim, Kyoung Heo, Min Kyung Chu

**Affiliations:** ^1^Department of Neurology, Incheon St. Mary's Hospital, College of Medicine, The Catholic University of Korea, Incheon, South Korea; ^2^Department of Neurology, Soonchunhyang University College of Medicine, Cheonan Hospital, Cheonan, South Korea; ^3^Department of Neurology, Seoul National University Bundang Hospital, Seoul National University College of Medicine, Seongnam, South Korea; ^4^Department of Neurology, Gangnam Severance Hospital, Yonsei University College of Medicine, Seoul, South Korea; ^5^Department of Neurology, Severance Hospital, Yonsei University College of Medicine, Seoul, South Korea

**Keywords:** insomnia, depression, clinical presentation, prevalence, epidemiology

## Abstract

**Objective:** Insomnia and depression are prevalent disorders that often co-occur. This study aimed to investigate the impact of clinically significant insomnia symptoms on the prevalence and clinical presentation of clinically significant depressive symptoms and vice versa.

**Methods:** This study used data from the Korean Headache-Sleep Study (KHSS), a nationwide cross-sectional population-based survey regarding headache and sleep. Clinically significant insomnia symptoms were defined as Insomnia Severity Index (ISI) scores ≥ 10 and clinically significant depressive symptoms were defined as Patient Health Questionnaire-9 (PHQ-9) scores ≥ 10, respectively. We referred clinically significant insomnia symptoms and clinically significant depressive symptoms as insomnia symptoms and depressive symptoms, respectively.

**Results:** Of 2,695 participants, 290 (10.8%) and 116 (4.3%) were classified as having insomnia and depressive symptoms, respectively. The prevalence of depressive symptoms was higher among participants with insomnia symptoms than in those without insomnia symptoms (25.9 vs. 1.7%, respectively, *P* < 0.001). Among participants with depressive symptoms, the PHQ-9 scores were not significantly different between participants with and without insomnia symptoms (*P* = 0.124). The prevalence of insomnia symptoms was significantly higher among participants with depressive symptoms than in those without depressive symptoms (64.7 vs. 8.3%, respectively, *P* < 0.001). The ISI scores were significantly higher among participants with insomnia and depressive symptoms than in participants with insomnia symptoms alone (*P* < 0.001).

**Conclusions:** Participants with depressive symptoms had a higher risk of insomnia symptoms than did those without depressive symptoms. The severity of depressive symptoms did not significantly differ based on insomnia symptoms among participants with depressive symptoms; however, the severity of insomnia symptoms was significantly higher in participants with depressive symptoms than in those without depressive symptoms.

## Introduction

Depression is a common mental health disorder that affects approximately 4.4% of the global population (1). Owing to its disabling symptoms and comorbidities, individuals with depression experience an increased risk of functional impairment and decreased quality of life (2). According to the Global Burden of Diseases Study 2015, depression was ranked as the third leading cause of disability among 310 disorders and injuries (3).

Insomnia is also a prevalent disorder that affects 9–15% of the general population (4). Insomnia has significant negative effects on various aspects of human functioning and is associated with greater disability at work, school, and home (5). Furthermore, individuals with insomnia reportedly use more health care services (6).

In addition to being common disorders that induce significant disability, depression, and insomnia are significantly associated with each other. Individuals with depression often report insomnia, which is an important symptom of depression. Furthermore, insomnia was included as a symptom in the diagnostic criteria of major depressive disorder (MDD) in the Diagnostic and Statistical Manual of Mental Disorders (DSM)-5 (7). Depression is reportedly more prevalent among those with insomnia than among individuals without insomnia (8). Longitudinal studies have shown that individuals with insomnia have a higher risk of developing depression, while individuals with depression have a higher risk of developing insomnia (9, 10). The existence of a bidirectional comorbidity suggests a shared pathophysiological mechanism between depression and insomnia (11).

Although a significant association between the two disorders has been persistently reported based on their elevated prevalence or co-occurrence, the literature features few clinic-based studies of the impact of insomnia on the clinical presentation of depression or vice versa. A clinic-based study including 4,041 patients with MDD reported that 84.1% of patients with MDD had insomnia symptoms. These patients also had more severe depressive symptoms than did the patients without insomnia symptoms (8). Another clinic-based study on patients with depressive disorder showed that 93% of them reported insomnia symptoms. Patients with depression and more severe insomnia (high insomnia) had more severe depressive symptoms than did those with less severe insomnia (low insomnia) (12).

Cross-sectional studies can obtain extensive information on exposures and outcomes, require a short time to conduct, and can acquire snapshot data, including prevalence (13). Population-based studies can represent the status of disorders in the general population (14). Hence, population-based cross-sectional studies can provide insight into the relationships between various disorders in the general population and may identify the association between certain disorders at a given point. Nevertheless, to the best of our knowledge, there is no information on the impact of insomnia on the clinical presentation of depression in a cross-sectional population-based setting.

The gold standard method of diagnosing insomnia and depression is based on established diagnostic criteria (7, 15). Nevertheless, this strategy is difficult to implement in epidemiological studies; therefore, a previous study evaluated insomnia and depressive symptoms, as well as their association, with a validated questionnaire (10).

Based on the results of previous studies, we hypothesized that individuals with clinically significant depressive symptoms had a higher risk of having clinically significant insomnia symptoms than those without depressive symptoms, and that among individuals with clinically significant depressive symptoms, those with clinically significant insomnia symptoms would have more severe symptoms of depressive symptoms than those without clinically significant insomnia symptoms (8–10, 12, 16). This study was aimed to investigate the impact of insomnia symptoms on the prevalence and clinical presentation of clinically significant depressive symptoms and vice versa using data from a nationally representative sample.

## Materials and Methods

### Survey

This study used data from the Korean Headache-Sleep Study (KHSS). The KHSS was a nationwide, cross-sectional survey of headache and sleep among adults aged 19–69 years in the Republic of Korea. The survey included items for headache, sleep, and their covariates, including depressive symptoms. The design, methods, and process of the KHSS have been described in detail in previous studies (17). Briefly, the study used a two-stage clustered random sampling method for all territories in the Republic of Korea, except Jeju-do. Sixty basic administrative units (cities and counties) were selected from 15 administrative divisions, and sampling was conducted. Seven cities were classified as large cities, while the rest were classified as medium-to-small cities; the counties were classified as rural areas. A target sample number for each division and basic administrative unit was determined based on the population distribution of the Republic of Korea. The estimated sampling error was 1.8% with a 95% confidence interval (CI). The survey was performed via door-to-door visits and face-to-face interviews by trained interviewers using questionnaires. All the interviewers were employees of Gallup Korea and had previous experience in conducting social surveys. Data collection was performed from November 2011 to January 2012. The KHSS was approved by the institutional review board/ethics committee of Hallym University Sacred Heart Hospital (IRB No. 2011–I077). Written informed consent was obtained from all the participants.

### Assessment of Clinically Significant Insomnia Symptoms

In this study, we used the Insomnia Severity Index (ISI), which is a self-reporting tool to measure a patient's perception of the severity of his/her insomnia during the previous 2 weeks, to diagnose and assess the severity of clinically significant insomnia symptoms. The ISI comprises the following items: 1a, difficulties in sleep onset; 1b, difficulties in sleep maintenance; 1c, early awakening in the morning; 2, dissatisfaction with current sleep pattern; 3, interference of sleep problems with daily functioning; 4, noticeability of the impairments attributed to sleep problems; and 5, worries caused by sleep problems. Each item was rated on a five-point Likert scale of 0–4 (items 1a–c: 0 = no problem, 4 = very severe problem; item 2, 0 = very satisfied, 4 = very dissatisfied; items 3–5, 0 = not at all, 4 = very much) (18). Higher total scores indicate greater insomnia severity. A cutoff score of 10 was optimal (86.1% sensitivity and 87.7% specificity) for detecting insomnia cases in a previous epidemiological study of insomnia (19). The single item “non-refreshing sleep in the morning” (0 = not at all, 4 = very much) was also assessed as an additional parameter. This study refers to clinically significant insomnia symptoms as insomnia symptoms.

### Assessment of Clinically Significant Depressive Symptoms

Clinically significant depressive symptoms was evaluated by using the Patient Health Questionnaire-9 (PHQ-9), which is a self-reported measure of depressive symptoms comprising nine items matching the DSM-IV criteria of MDD: 1, little interest or pleasure in doing things; 2, feeling down, depressed, or hopeless; 3, trouble with sleep; 4, feeling tired or having little energy; 5, poor appetite or overeating; 6, feeling bad about yourself; 7, trouble concentrating on things; 8, moving or speaking too slowly or too fast; and 9, suicidal thoughts. Respondents were asked to rate each of the items on a scale of 0 (not at all) to 3 (nearly every day) on the basis of how frequently a symptom had bothered them over the last 2 weeks. The scores from each of the items were summed to give a total score ranging from 0 to 27 (20). A cut-off score of ≥10 on the summed-item score has been recommended as a method of screening for MDD in the Korean version of PHQ-9 (81.8% sensitivity and 89.9% specificity), with an excellent value (0.944) of the area under curve (21). This study refers to clinically significant depressive symptoms as depressive symptoms.

### Statistical Analyses

We evaluated the impact of insomnia symptoms on the prevalence of depressive symptoms by calculating the age- and sex-adjusted odds ratio (OR): the ratio of the odds of having depressive symptoms in participants with insomnia symptoms to the odds in participants without insomnia symptoms, as determined, using logistic regression analyses. To investigate the impact of insomnia symptoms on the clinical presentation of depressive symptoms, we compared the severity of depressive symptoms (PHQ-9 score) between participants with depressive symptoms who had and did not have insomnia symptoms using multiple linear regression analyses after adjusting for age and sex. For assessing the impact of depressive symptoms on the prevalence on insomnia symptoms, we determined the age- and sex-adjusted OR of having insomnia symptoms between participants with and without depressive symptoms. We evaluated the impact of depressive symptoms on the clinical presentation of insomnia symptoms by comparing the severity of insomnia (ISI score) between participants with insomnia symptoms who had and did not have depressive symptoms using multiple linear regression analyses after adjusting for age and sex. Since the prevalence and severity of insomnia and depressive symptoms significantly vary with age (22–24), we further evaluated the associations between insomnia and depressive symptoms by dividing the participants into age groups of 19–35, 36–52, and 53–69 years.

We set the level of significance for the two-tailed tests at 0.05. Statistical analyses were performed using IBM SPSS for Windows version 23.0 (IBM Corp., Armonk, NY, USA).

## Results

### Survey

**Figure 1:**
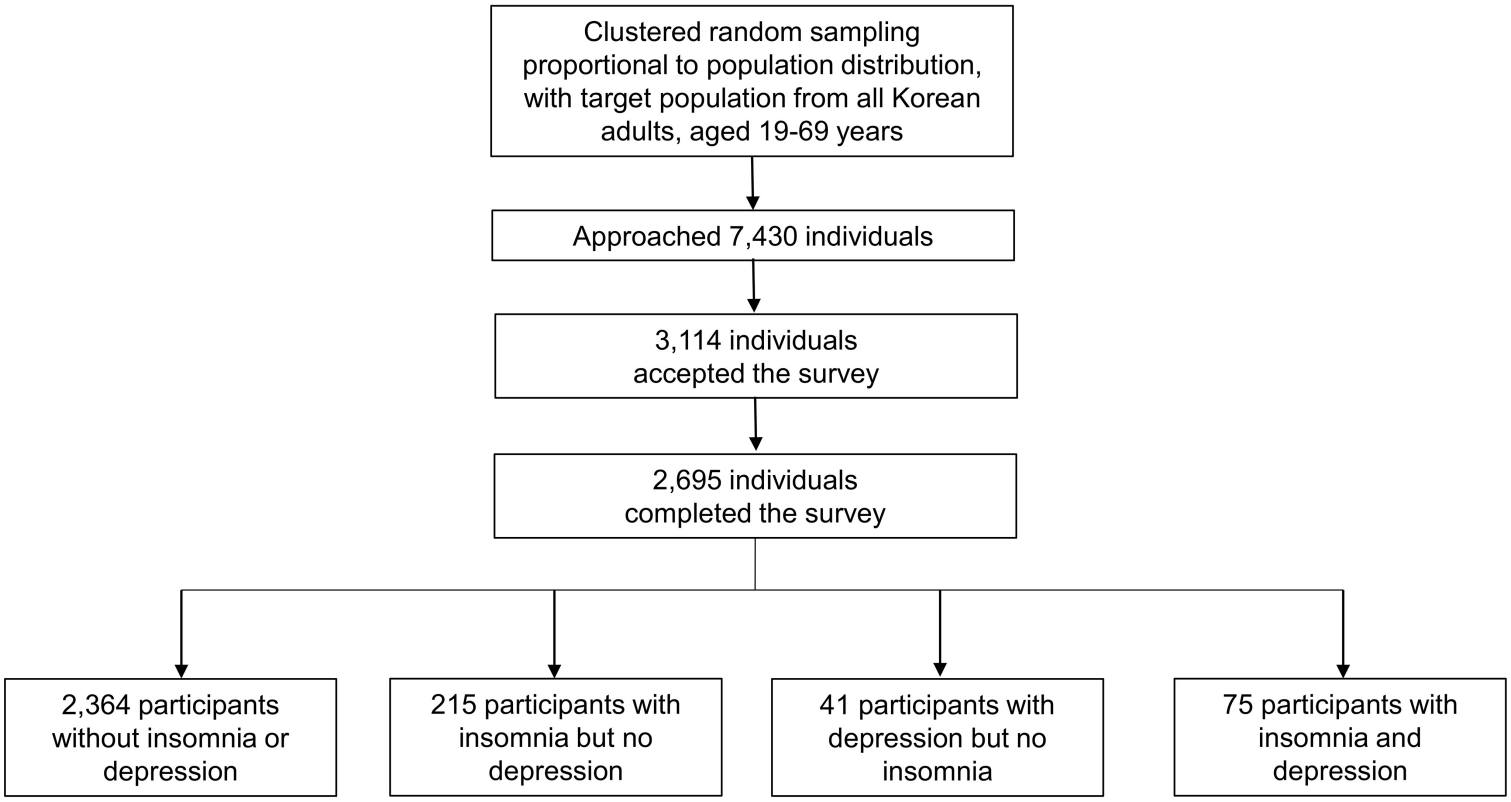
Flow chart depicting the participation of individuals in the Korean Headache-Sleep Study.

The interviewers contacted 7,430 individuals, and 3,114 of them agreed to the survey. Of those who initially agreed, 419 individuals withdrew from the survey. In total, 2,695 participants completed the survey without any missing data, except education level (cooperation rate of 36.3%; Figure 1). The distributions of age, sex, size of residential area, and education level were not significantly different from those of the general population of the Republic of Korea (Table 1) (25).

**Table 1:** Sociodemographic characteristics of the survey participants, total population in the Republic of Korea, and survey cases identified as having depressive symptoms and insomnia symptoms.

	**Survey participants**	**Total population (25)**	***P*-value**	**Depressive symptoms^†^**	**Insomnia symptoms^‡^**
	***N* (%)**	***N* (%)**		***N*, % (95% CI)**	***N*, % (95% CI)**
**Sex**					
Male	1,345 (49.9)	17,584,365 (50.6)	0.854^*^	43, 3.2 (2.3–4.2)	117, 8.7 (7.2–10.2)
Female	1,350 (50.1)	17,198,350 (49.4)		73, 5.4 (4.2–6.6)	173, 12.8 (11.0–14.6)
**Age (in years)**					
19–29	542 (20.1)	7,717,947 (22.2)	0.917^*^	23, 4.2 (2.5–5.8)	59, 10.9 (8.3–13.5)
30–39	604 (22.4)	8,349,487 (24.0)		32, 5.3 (4.6–7.3)	53, 8.8 (6.5–11.0)
40–49	611 (22.6)	8,613,110 (24.8)		24, 4.0 (2.5–5.5)	66, 10.8 (8.3–13.3)
50–59	529 (19.6)	6,167,505 (17.7)		22, 4.2 (2.5–6.0)	63, 11.9 (9.1–14.7)
60–69	409 (15.2)	3,934,666 (11.3)		15, 3.7 (2.0–5.5)	49, 12.0 (8.8–15.1)
**Size of residential area**					
Large cities	1,248 (46.3)	16,776,771 (48.2)	0.921^*^	57, 4.6 (3.4–5.7)	136, 10.9 (9.2–12.6)
Medium-to-small cities	1,186 (44.0)	15,164,345 (43.6)		47, 4.0 (2.9–5.1)	125, 10.5 (8.8–12.3)
Rural areas	261 (9.7)	2,841,599 (8.2)		12, 4.6 (2.1–7.3)	29, 11.1 (7.3–14.9)
**Education level**					
Middle school or less	393 (14.6)	6,608,716 (19.0)	0.752^*^	20, 5.1 (3.0–7.4)	62, 15.8 (12.2–19.4)
High school	1,208 (44.8)	15,234,829 (43.8)		49, 4.1 (3.0–5.2)	116, 9.6 (7.9–11.3)
College or more	1,068 (39.6)	12,939,170 (37.2)		47, 4.4 (3.2–5.7)	109, 10.2 (8.4–12.0)
Not responded	26 (0.1)				3, 11.5 (0.0–24.7)
**Total**	2,695 (100.0)	34,782,715 (100.0)		116, 4.3 (3.6–5.1)	290, 10.8 (9.6–11.9)

* *Comparison of sex, age, size of residential area, and education level distributions between the sample in this study and the total population of the Republic of Korea. N, number; CI, confidence interval*.

† *Patient Health Questionnaire-9 score ≥ 10*.

‡ *Insomnia Severity Index score ≥ 10*.

### Prevalence of Insomnia and Depressive Symptoms

Of the 2,695 participants who completed the survey, 290 (10.8%) had an ISI ≥ 10 and were classified as having insomnia symptoms. One hundred and sixteen had a PHQ-9 score ≥ 10 and were classified as having depressive symptoms (Table 1).

### Impact of Insomnia Symptoms on the Prevalence of Depressive Symptoms

**Figure 2:**
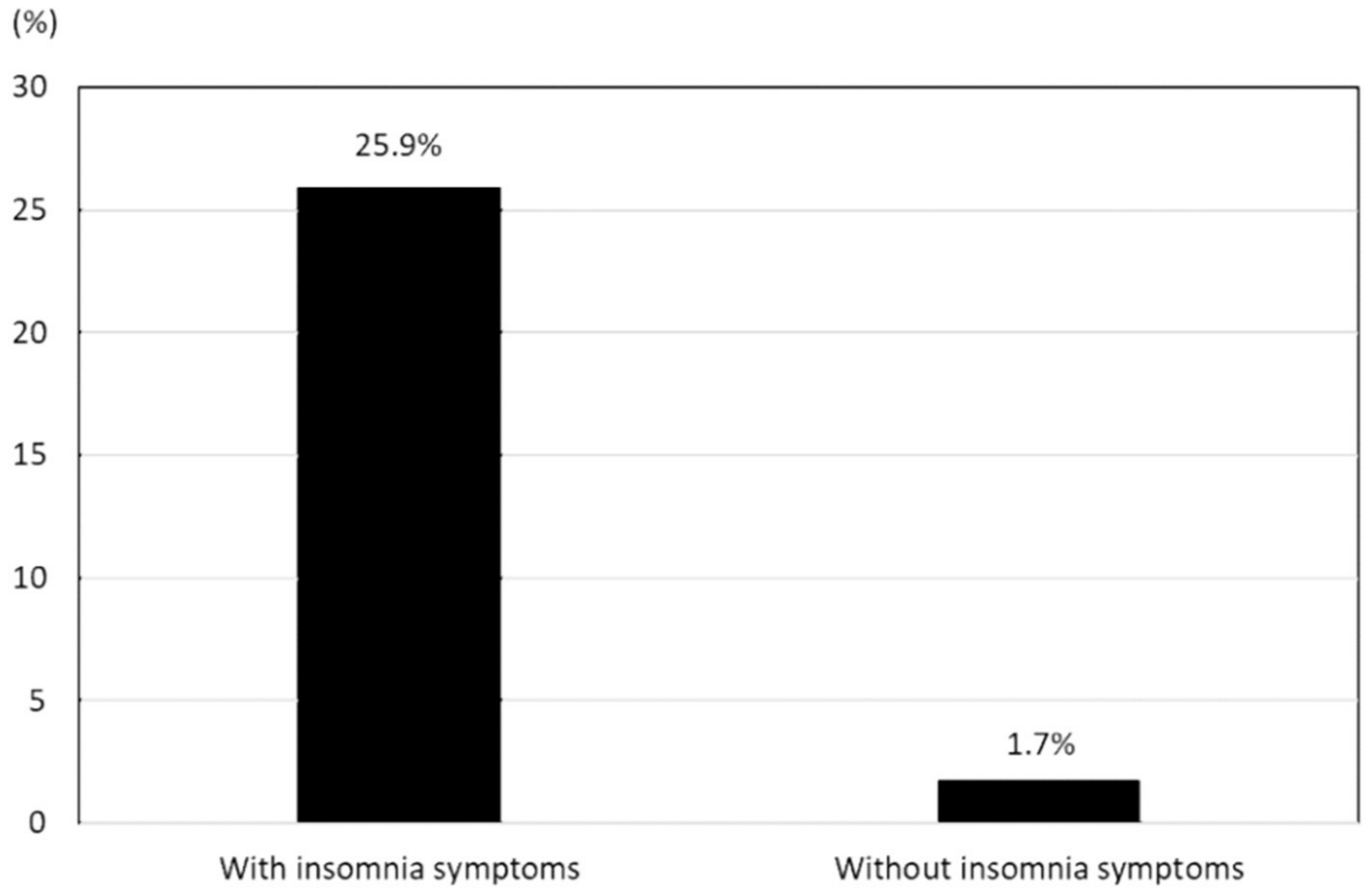
Prevalence of depressive symptoms in participants with insomnia symptoms and those without insomnia symptoms.

Among 290 participants with insomnia symptoms, 75 were also classified as having depressive symptoms. Additionally, 41 of the 2,405 individuals without insomnia symptoms had PHQ-9 scores indicating depressive symptoms. The prevalence of depressive symptoms was significantly higher among participants with insomnia symptoms than among those without insomnia symptoms (25.9 vs. 1.7%, *P* < 0.001; Figure 2). The age- and sex-adjusted OR for the prevalence of depressive symptoms with respect to the presence or absence of insomnia symptoms was 21.8 (95% CI = 14.1–33.7).

### Impact of Insomnia Symptoms on the Clinical Presentation of Depressive Symptoms

Among participants with depressive symptoms, the total PHQ-9 scores were not significantly different between individuals with and without insomnia symptoms (13.0 [10.0–17.0] vs. 12.0 [11.0–13.5], *P* = 0.124). Of the nine PHQ-9 items, the “trouble with sleep” score was significantly higher in participants with both depressive and insomnia symptoms than in participants with depressive symptoms, but without insomnia symptoms. Scores on the other items of the PHQ-9 did not significantly differ between the two groups (Table 2).

**Table 2:** Total and item scores of the PHQ-9 among individuals with depressive symptoms according to the presence of insomnia symptoms.

	**Individuals with depressive and insomnia symptoms, *N* = 75**	**Individuals with depressive symptoms, but no insomnia symptoms, *N* = 41**	*P*-value^*^
	**Median (25–75%)**	**Median (25–75%)**	
1. Little interest or pleasure in doing things	2.0 (1.0–2.0)	2.0 (1.0–2.0)	0.561
2. Feeling down, depressed or hopeless	2.0 (1.0–2.0)	2.0 (1.0–2.0)	0.352
3. Trouble with sleep	2.0 (2.0–3.0)	1.0 (2.0–3.0)	<0.001
4. Feeling tired or having little energy	2.0 (2.0–3.0)	2.0 (1.0–2.5)	0.028
5. Poor appetite or overeating	2.0 (1.0–3.0)	2.0 (1.0–3.0)	0.795
6. Feeling bad about yourself	1.0 (0.0–2.0)	2.0 (0.5–2.0)	0.951
7. Trouble concerning on things	1.0 (0.0–2.0)	1.0 (1.0–2.0)	0.512
8. Moving or speaking too slow or too fast	1.0 (0.0–2.0)	0.0 (0.0–1.0)	0.635
9. Suicidal thoughts	1.0 (0.0–2.0)	1.0 (0.0–2.0)	0.565
**Total**	13.0 (10.0–17.0)	12.0 (11.0–13.5)	0.076

*N, number; PHQ-9, Patient Health Questionnaire-9*.

* *Multiple linear regression analyses adjusting age and sex*.

### Impact of Depressive Symptoms on the Prevalence of Insomnia Symptoms

**Figure 3:**
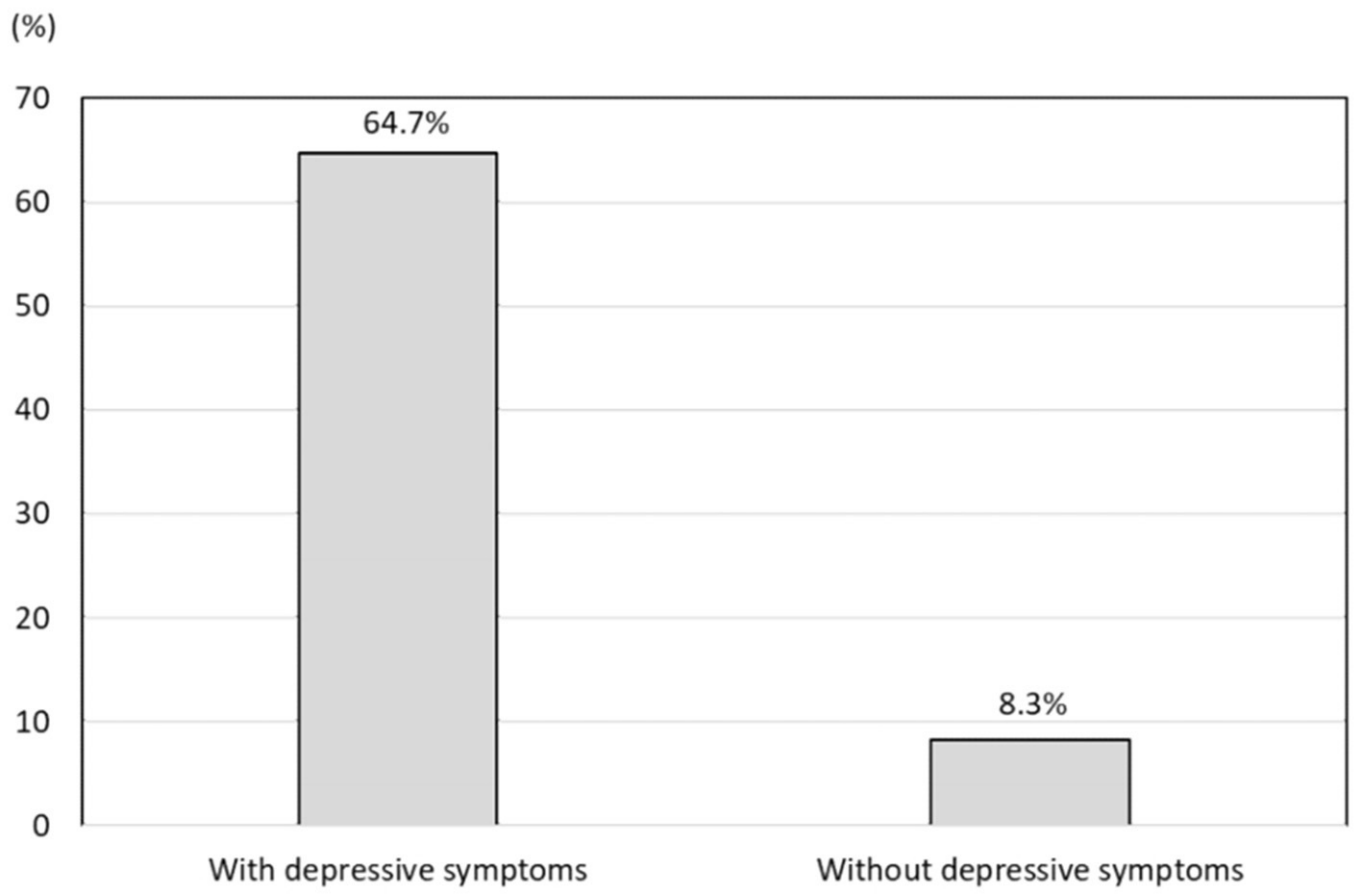
Prevalence of insomnia symptoms in participants with depressive symptoms and those without depressive symptoms.

Among 116 participants with depressive symptoms, 75 (64.7%) were also classified as having insomnia symptoms. In addition, 215 (8.3%) of the 2,579 participants without depressive symptoms were classified as having insomnia symptoms (Figure 3). The prevalence of insomnia symptoms among participants with depressive symptoms was significantly higher than that among participants without depressive symptoms (*P* < 0.001). The age- and sex-adjusted OR for the prevalence of insomnia symptoms with respect to the presence or absence of depressive symptoms was 21.5 (95% CI = 13.9–33.2).

### Impact of Depressive Symptoms on the Clinical Presentation of Insomnia Symptoms

The total ISI scores were significantly higher among participants with insomnia and depressive symptoms than among participants with insomnia, but without depressive symptoms (17.0 [12.0–21.0] vs. 12.0 [11.0–15.0], *P* < 0.001). Additional analyses of the seven ISI items demonstrated that scores on all items, except “early awakening in the morning,” were significantly higher in the participants with insomnia and depressive symptoms than in those with insomnia but without depressive symptoms. Moreover, the score for the additional item “non-refreshing sleep in the morning” was significantly higher among participants with insomnia and depressive symptoms than among those with insomnia symptoms alone (Table 3).

**Table 3:** Total and item scores of the ISI among individuals with insomnia symptoms according to the presence of depressive symptoms.

	**Individuals with insomnia and depression symptoms, *N* = 75**	**Individuals with insomnia symptoms but no depressive symptoms, *N* = 215**	*P*-value^*^
	**Median (25–75%)**	**Median (25–75%)**	
Falling asleep	3.0 (2.0–4.0)	2.0 (1.0–3.0)	0.001
Staying asleep	3.0 (1.0–3.0)	2.0 (1.0–3.0)	<0.001
Early awakening	2.0 (1.0–3.0)	2.0 (1.0–3.0)	0.089
Non-refreshing sleep in the morning	3.0 (3.0–4.0)	2.0 (1.0–3.0)	<0.001
Satisfaction with current sleep pattern	4.0 (4.0–5.0)	4.0 (3.0–4.0)	<0.001
Interference with daily functioning	3.0 (2.0–4.0)	3.0 (2.0–4.0)	0.004
Impaired quality of life	3.0 (2.0–4.0)	3.0 (2.0–3.0)	0.001
Worry about current sleep problems	3.0 (2.0–4.0)	3.0 (2.0–3.0)	<0.001
**Total**	17.0 (12.0–21.0)	12.0 (11.0–15.0)	<0.001

*N, number; ISI, Insomnia Severity Index*.

* *Multiple linear regression analyses after adjusting for age and sex*.

### Impact of Insomnia Symptoms on the Prevalence and Clinical Presentation of Depressive Symptoms According to Age

The prevalence of depressive symptoms among participants with insomnia symptoms was significantly higher than among participants without insomnia symptoms in all age groups. Among the participants with depressive symptoms, the total PHQ-9 scores were not significantly different between participants with and without insomnia symptoms in all age groups (Supplementary Table 1A and Supplementary Table 2A).

### Impact of Depressive Symptoms on the Prevalence and Clinical Presentation of Insomnia Symptoms at Different Age Groups

The prevalence of insomnia symptoms among participants with depressive symptoms was significantly higher than among participants without insomnia symptoms in all age groups. Among the participants with insomnia symptoms, the total ISI scores were significantly higher among participants with depressive symptoms than those without depressive symptoms in all age groups (Supplementary Table 1B and Supplementary Table 2B).

## Discussion

The key findings of this study were as follows: 1. The prevalence of insomnia and depressive symptoms in the adult population of the Republic of Korea was 10.8 and 4.3%, respectively. 2. The prevalence of depressive symptoms was higher in the participants with insomnia symptoms than in the participants without insomnia symptoms; nevertheless, the severity of depressive symptoms did not significantly differ between participants with depressive and insomnia symptoms and those with depressive symptoms, but without insomnia symptoms. 3. The prevalence of insomnia symptoms was higher in participants with depressive symptoms than in participants without depressive symptoms; moreover, the severity of insomnia symptoms was higher in the participants with insomnia and depressive symptoms than in the participants with insomnia symptoms, but without depressive symptoms.

Based on the findings in this study, we accepted the hypothesis that participants with insomnia symptoms had an increased risk of depressive symptoms. Nevertheless, we had to reject the hypothesis that the severity of the depressive symptoms was higher in the participants with depressive and insomnia symptoms than in the participants with depressive symptoms alone.

Although the close association between insomnia and depression has been consistently reported in cross-sectional and longitudinal studies, information on the impact of insomnia symptoms on the prevalence and clinical presentation of depressive symptoms is limited. This study is the first report to compare the impact of insomnia symptoms on the clinical presentation of depressive symptoms with the impact of depressive symptoms on the clinical presentation of insomnia symptoms in a population setting.

Two previous clinical studies have assessed the severity of depressive symptoms according to the presence of insomnia. The STARD^*^D study, which enrolled patients with depression in clinics, reported that the depressive symptoms in patients with insomnia symptoms were more severe than that in those without insomnia symptoms (8). The CRESCEND study of patients with depression revealed that patients with depression and high insomnia had more severe depressive symptoms than did patients with depression and low insomnia (12). These findings contradicted the present finding that the severity of depressive symptoms in participants with depression did not change according to the severity of insomnia symptoms. One possible explanation for the discrepancy between the present and previous findings may be attributed to the study settings. The two previous studies were conducted in clinical settings, while this was a population-based study. Different study setting between clinic-based and population-based studies may result in different outcomes.

One explanation for the impact of insomnia symptoms on depressive symptoms and vice versa may involve possible differences in a shared mechanism underlying each condition. Reductions in total sleep time, sleep efficiency, and slow wave sleep time have been observed in individuals with depression as well as in individuals with insomnia (26, 27). However, significant changes in rapid eye movement (REM) sleep, including a decrease in REM latency, an increase in the proportion of REM sleep, and elevated REM density, were observed in individuals with depression, whereas there was no change in these parameters in those with primary insomnia (28–30). These findings suggest that there is a difference between depression and insomnia with respect to the physiological mechanisms of REM sleep. Dysregulation of REM sleep and depression were related to dysfunction of the monoaminergic system (31). The depletion of monoamines by the administration of reserpine induced depression and acceleration of REM sleep (32). An increase in extracellular 5-hydroxytryptamine (5-HT) improved depression and inhibited REM sleep (33). Studies on serotonergic dysfunction in primary insomnia revealed conflicting results. 5-hydroxytryptamine neurons promoted wakefulness, and insomnia caused by 5-HT depletion was due to hypothermia rather than a sleep-inducing effect (34, 35). Selective serotonin reuptake inhibitors were effective in patients with insomnia who did not have depression (36). Differences in the hypothalamus-pituitary-adrenal (HPA) system have also been reported between patients with depression and those with insomnia: specifically, elevated activity of the HPA system in patients with insomnia (37–39). Urinary free cortisol (UFC) has been used as an indicator of the activity of HPA system (40). Patients with insomnia showed elevated UFC levels, and their level of 24-h UFC was positively correlated with wake time (37). Among individuals with depression, elevated HPA system activity was also observed (41–43). Nevertheless, findings regarding UFC levels have been inconsistent. Some studies reported no significant differences between patients with depression and those without, while others found increased UFC levels in patients with depression (44, 45). These findings suggest that depression and primary insomnia share a pathophysiological mechanism, but that some aspects are distinct from one another.

Among the PHQ-9 items, scores on the items for “trouble with sleep” and “feeling tired or have little energy” were higher among participants with depression and insomnia symptoms than among participants with depressive symptoms alone. This may be due to the characteristics of the items. The PHQ-9 was constructed based on the nine criteria for the diagnosis of MDD according to the DSM-IV, which included sleep problems as one of the criteria (20). Consequently, participants with depressive and insomnia symptoms more frequently responded positively to the item “trouble with sleep” than did those with depression but without insomnia symptoms. Hence, the higher score for the item “feeling tired or have little energy” among participants with depressive and insomnia symptoms may be attributed to the nature of the item. Feeling tired and having little energy is key symptoms for both insomnia and depression (46, 47). Therefore, individuals with depression on feeling tired or having little energy had more depressive symptoms; they might have more severe depressive symptoms (20). Nevertheless, the remaining seven items, which were not directly related to sleep, did not significantly differ based on the status of insomnia symptoms.

In a prior study, the ISI was evaluated for its validity and reliability for classifying insomnia through mail in 959 community-based individuals. Based on the responses and analyses using receiver operating curves, a cut-off value of 10 showed optimal results (19). The ISI showed a good correlation with sleep diaries and polysomnographic results among 183 individuals with insomnia. Furthermore, the ISI showed a good sensitivity to detect clinical improvements after treatment (19). The items of the ISI comprise insomnia symptoms and their consequences (18). In these studies, the insomnia prevalence based on insomnia symptoms and daytime consequences mostly ranged from 9 to 15%, which was similar to that in this study (4).

The prevalence of depressive symptoms in this study was 4.3%, which was comparable to those observed in previous studies. Studies using the Composite International Diagnostic Interview, Diagnostic Interview Schedule, and DSM-III to classify depression showed that the prevalence of depression in the Republic of Korea ranged from 1.3 to 4.0% (23, 48, 49). Among studies using the PHQ-9, the prevalence of depressive symptoms ranged between 4.2 and 6.4%.

This study showed the point prevalence of insomnia symptoms as 10.8%. Although a variety of diagnostic criteria or instruments were used, previous reports on the prevalence of insomnia symptoms have ranged widely from 5.0 to 22.8% (50, 51). The prevalence of insomnia symptoms in this study is within this previously reported range. Furthermore, a Canadian population-based study using the ISI reported the prevalence of insomnia observed as 7.4% (52), which was similar to our study. The similarity in the prevalence of depressive and insomnia symptoms between this study and previous studies suggests the reliability of the assessment of depression and insomnia symptoms in the present study.

The findings of this study may be helpful in the management of insomnia and depression. If an individual is diagnosed with insomnia or depression, an assessment of the other disease is urgently needed since the risk of comorbidity is remarkably high. In addition to providing insight into the relationship between the two disorders, the results of our study also provide additional prospects in the understanding of the nature of insomnia and depression.

This study found that 64.7% of participants with depressive symptoms had insomnia symptoms. It has been previously reported that approximately 50–70% of individuals with depression experienced insomnia (53, 54). Thus, depression could be classified according to the presence of insomnia (depression with insomnia vs. depression without insomnia) considering the close relationship of the two disorders. Similarly, insomnia could be classified as insomnia with depression and insomnia without depression (depressive insomnia vs. non-depressive insomnia). Our findings would also be useful in medical policy making. The severity of the disease is an important factor in determining the priority of medical care (55). If an individual has both insomnia and depression, the symptoms of insomnia experienced would be more severe than those experienced in insomnia without depression; therefore, individuals with both insomnia and depression should be considered as a priority for treatment of insomnia. The severity of depression in individuals with insomnia is less likely to be more severe than their counterparts without insomnia. Nevertheless, both depression and insomnia are serious disorders, and the proper treatment of accompanying insomnia is urgently needed in individuals with depression.

This was a cross-sectional study and could not provide definitive information on the casual association between depressive and insomnia symptoms but could provide an insight to infer causations (56). The present study observed that the participants with insomnia had an increased prevalence of depression and those with depression had an increased prevalence of insomnia. These findings suggest a possibility of an increased risk of developing depression in individuals with insomnia and vice versa. As expected, a significant bidirectional association between insomnia and depressive symptoms has been reported in longitudinal studies (9–11, 57). For the impact of depressive symptoms on the clinical presentation of insomnia symptoms, we can hypothesize that individuals with insomnia symptoms combined with depressive symptoms would develop more severe insomnia symptoms than counterparts without depressive symptoms in follow-up. In contrast, individuals with depressive symptoms combined with insomnia symptoms can be hypothesized that their depressive symptoms may not significantly differ from those without insomnia symptoms in follow-up. Nevertheless, such associations have not been reported and further longitudinal studies will confirm the complex relationship between insomnia and depressive symptoms.

This study has some limitations. First, we did not assess the use of medications for insomnia or depression. Insomnia and depression are common disorders in the general population and a significant proportion of individuals use medications for depression and insomnia (58, 59). Medications for depression and insomnia may affect the prevalence and symptom severity of both disorders (60, 61). Second, this study used data from the KHSS, which were collected in 2011 and 2012. Therefore, this study provides information on the status and association between depression and insomnia as of 9 years ago. Nevertheless, as the prevalence of depression and insomnia has remained stable over the past 9 years in the Republic of Korea (49, 62), the findings of this study may still be valid. Lastly, we evaluated insomnia symptoms based on the ISI rather than using objective measures such as actigraphy or polysomnography. Insomnia symptoms could occur due to various causes including restless legs syndrome, obstructive sleep apnea, sleep-related movement disorder, and so forth (63). Therefore, the insomnia symptoms reported by the respondents in this study were attributable to various conditions.

Our study also has several strengths. First, this study used a population-based sample with a large sample size that used two-stage clustered sampling methods proportional to the population distribution of the Republic of Korea. In addition, the estimated sampling error was low. Consequently, we could accurately investigate the impact of insomnia on the prevalence and clinical characteristics of depression in a population-based setting. Furthermore, we investigated depressive and insomnia symptoms using validated questionnaires, which have shown high sensitivity and specificity. Given the similarities in the prevalence of depressive and insomnia symptoms with previous studies and use of valid questionnaires, we are assured that we accurately evaluated depressive and insomnia symptoms in our study. Lastly, this study explored the impact of insomnia symptoms on depressive symptoms along with the impact of depressive symptoms on insomnia symptoms, which has rarely been studied previously.

## Conclusions

In conclusion, both depressive and insomnia symptoms were prevalent conditions in a general population-based sample from the Republic of Korea. Participants with depressive symptoms showed a higher prevalence of insomnia symptoms than participants without depressive symptoms. However, the severity of depressive symptoms was not significantly affected by the presence of insomnia symptoms. The prevalence of depressive symptoms was higher among participants with insomnia symptoms than among those without insomnia symptoms. Participants with both insomnia and depressive symptoms showed more severe insomnia symptoms than participants with insomnia symptoms alone.

## Data Availability Statement

The data used in the present study are available from the corresponding author on reasonable request.

## Ethics Statement

The studies involving human participants were reviewed and approved by Hallym University Sacred Heart Hospital Ethics committee. The patients/participants provided their written informed consent to participate in this study. Written informed consent was obtained from the individual(s) for the publication of any potentially identifiable images or data included in this article.

## Author Contributions

YC and MC prepared the concept and design of the research study. YC performed the analyses and wrote the original version of the manuscript. MC supervised the analyses and critically revised the manuscript. KY, C-HY, W-JK, and KH participated in the collection of data, revised the manuscript for intellectual content. All authors have reviewed the process of data analysis, writing of the manuscript, and approved the final article.

## Conflict of Interest

MC was a site investigator for a multi-center trial sponsored by Otsuka Korea, Novartis, International AG, and Eli Lilly and Co. He functioned as an advisory member for Teva, and received lecture honoraria from Allergan Korea, Handok-Teva, and Yuyu Pharmaceutical Company over the past 24 months. He received grants from the Yonsei University College of Medicine (2018-32-0037) and National Research Foundation of Korea (2019R1F1A1053841). The remaining authors declare that the research was conducted in the absence of any commercial or financial relationships that could be construed as a potential conflict of interest.

## Publisher's Note

All claims expressed in this article are solely those of the authors and do not necessarily represent those of their affiliated organizations, or those of the publisher, the editors and the reviewers. Any product that may be evaluated in this article, or claim that may be made by its manufacturer, is not guaranteed or endorsed by the publisher.
