# Supplementary material for: Impact of Insomnia Symptoms on the Clinical Presentation of Depressive Symptoms: A Cross-Sectional Population Study
Source: Front Neurol. 2021 Aug 9;12:716097. doi: 10.3389/fneur.2021.716097 (PMC8381020; doi:10.3389/fneur.2021.716097)
Supplement: Supplementary file 1 [file Data_Sheet_1.docx]

**Supplementary Table 1.** Impact of insomnia symptoms on the prevalence of depressive symptoms (A) and impact of depressive symptoms on the prevalence of insomnia symptoms at different age groups (B) at different age groups.

(A)

| **Age groups** | **With insomnia symptoms, N (%)** | **Without insomnia symptoms, N(%)** | ***P*-value** |
| --- | --- | --- | --- |
| 19-35 | 27/92 (29.3) | 17/772 (2.2) | <0.001 |
| 36-52 | 26/107 (24.3) | 19/971 (2.0) | <0.001 |
| 53-69 | 22/91 (24.2) | 5/662 (0.8) | <0.001 |

(B)

| **Age groups** | **With depressive symptoms, N (%)** | **Without depressive symptoms, N(%)** | ***P*-value** |
| --- | --- | --- | --- |
| 19-35 | 27/44 (61.4) | 65/820 (7.9) | <0.001 |
| 36-52 | 26/45 (57.8) | 81/1033 (7.8) | <0.001 |
| 53-69 | 22/27 (81.5) | 69/726 (75.8) | <0.001 |

Abbreviations: N, number

**Supplementary Table 2.** Impact of insomnia symptoms on the severity of depressive symptoms in participants with depressive symptoms (A) and impact of depressive symptoms on the severity of insomnia symptoms in participants with insomnia symptoms (B) at different age groups

(A)

| **Age groups** | **With insomnia symptoms, PHQ-9 score, median and IQR** | **Without insomnia symptoms, PHQ-9 score, median and IQR** | ***P*-value** |
| --- | --- | --- | --- |
| 19-35 | 13.0 (10.0-19.0) | 13.0 (10.5-14.5) | 0.600 |
| 36-52 | 15.0 (10.0-17.0) | 12.0 (11.0-12.0) | 0.133 |
| 53-69 | 13.0 (10.0-15.0) | 11.0 (10.0-21.0) | 0.880 |

(B)

| **Age groups** | **With depressive symptoms, ISI score, median and IQR** | **Without depressive symptoms, ISI score, median and IQR** | ***P*-value** |
| --- | --- | --- | --- |
| 19-35 | 14.0 (12.0-18.0) | 11.0 (10.0-14.0) | 0.008 |
| 36-52 | 17.0 (13.0-24.5) | 12.0 (11.0-15.0) | 0.001 |
| 53-69 | 17.0 (13.75-23.0) | 13.0 (11.5-16.0) | <0.001 |

Abbreviations: ISI, Insomnia Severity Index; PHQ-9, Patient Health Questionnaire-9; IQR, interquartile range
